# Surface Acoustic Wave Hydrogen Sensors Based on Nanostructured Pd/WO_3_ Bilayers

**DOI:** 10.3390/s18113636

**Published:** 2018-10-26

**Authors:** Dana Miu, Ruxandra Birjega, Cristian Viespe

**Affiliations:** Laser Department, Plasma and Radiation Physics, National Institute for Laser, Atomistilor # 409, 077125 Bucharest-Magurele, Romania; dana.miu@inflpr.ro (D.M.); ruxandra.birjega@inflpr.ro (R.B.)

**Keywords:** surface acoustic wave, Pd, WO_3_, bilayer, thin film, nanostructure, hydrogen detection, pulsed laser deposition, gas sensor

## Abstract

The effect of nanostructure of PLD (Pulsed Laser Deposition)-deposited Pd/WO_3_ sensing films on room temperature (RT) hydrogen sensing properties of SAW (Surface Acoustic Wave) sensors was studied. WO_3_ thin films with different morphologies and crystalline structures were obtained for different substrate temperatures and oxygen deposition pressures. Nanoporous films are obtained at high deposition pressures regardless of the substrate temperature. At lower pressures, high temperatures lead to WO_3_ c-axis nanocolumnar growth, which promotes the diffusion of hydrogen but only once H_2_ has been dissociated in the nanoporous Pd layer. XRD (X-ray Diffraction) analysis indicates texturing of the WO_3_ layer not only in the case of columnar growth but for other deposition conditions as well. However, it is only the predominantly c-axis growth that influences film sensing properties. Bilayers consisting of nanoporous Pd layers deposited on top of such WO_3_ layers lead to good sensing results at RT. RT sensitivities of 0.12–0.13 Hz/ppm to hydrogen are attained for nanoporous bilayer Pd/WO_3_ films and of 0.1 Hz/ppm for bilayer films with a nanocolumnar WO_3_ structure. SAW sensors based on such layers compare favorably with WO_3_-based hydrogen detectors, which use other sensing methods, and with SAW sensors with dense Pd/WO_3_ bilayers.

## 1. Introduction

The development of sensitive gas sensors is an active research domain due to the serious safety hazard problems implied by its use [1,2,3,4]. WO_3_ is a wide band gap n-type metal oxide semiconductor, which is used for detecting various gases [2]. In the case of hydrogen sensing, satisfactory results are obtained only when combined with catalytic metals such as Pt [5] or Pd [6,7], which lead to the dissociation of H_2_ and generates H^+^ ions and electrons, which diffuse into the WO_3_ layer. Sensing is achieved through various mechanisms such as gasochromic [8], resistive [9], or Surface Acoustic Waves (SAW) [10].

The SAW sensing mechanism is based on the perturbation of surface acoustic wave propagation due to changes in mass and the mechanical or electrical properties of the sensing layer in the presence of a gas. Bilayer sensing layers can, in certain conditions, lead to an improved sensor response through increased acoustoelectric effects [11,12]. This is achieved by ensuring that the conductivity of the sensor is in the optimal range and by optimizing film thicknesses [13,14]. In the case of Pd/WO_3_ layers, however, although SAW sensor results are better than the single WO_3_ due to the acoustoelectric interaction added to the mass effect (which is small for H_2_), the results for dense Pd/WO_3_ bilayers are not satisfactory at temperatures close to RT (room temperature) [10]. Studies on the effect of film morphology and crystalline orientation on the sensing properties of WO_3_ and Pd/WO_3_ films have only been studied in relation to resistive or gasochromic responses to gas and not in the case of SAW [6,8,9]. These studies have shown that sensing properties can be optimized through the control of the structural properties of the film. However, relatively high sensor operating temperatures are required for all types of Pd/WO_3_–based sensors.

We have studied the effect of the nanostructure of pulsed-laser deposited Pd/WO_3_ bilayer sensing films on the H_2_ sensing properties of SAW sensors. WO_3_ films with different morphologies and crystalline structures were obtained in different Pulsed Laser Deposition (PLD) conditions depending on the parameters used (deposition temperature, pressure). SAW sensor properties of films consisting of thin nanoporous Pd on WO_3_ layers towards H_2_ at various concentrations were determined. The combination of nanoporous Pd, which is efficient in dissociating H_2_, and WO_3_ films of various structures (nanoporous, nanocolumnar) leads to good sensing results at room temperature. The resulting SAW sensors compare favorably with WO_3_-based hydrogen detectors, which use other sensing methods, and with SAW sensors with dense Pd/WO_3_ bilayers.

## 2. Materials and Methods

WO_3_ thin films and Pd/WO_3_ multilayers were deposited by PLD (pulsed laser deposition) using a Nd-YAG laser (EKSPLA NL301HT, Ekspla, Vilnius, Lithuania) with a 5 ns pulse duration at a pulse repetition rate of 10 Hz and an emission wavelength of 532 nm. The SAW sensor sensitive films were deposited onto ST-X quartz substrates positioned 40 mm from the WO_3_ and Pd targets in a vacuum chamber equipped with a gas flow and pressure control system. The targets were placed on computer-controlled x-y tables, which allow multilayer deposition as well as target scanning during deposition to avoid target erosion. This leads to an increased surface roughness and a lack of uniformity of the films. The temperature of the substrate heater was controlled by using a Model 101303-22 Heat Wave Labs temperature controller.

The WO_3_ films were deposited at various temperatures (RT, 300 °C, 450 °C, 600 °C) in an oxygen atmosphere at various pressures (150, 300, 500 mTorr) in order to obtain films with different morphologies and crystalline structures. An energy of 120 mJ/pulse was used for ablation. The Pd films were deposited on top of the WO_3_ films in an Ar atmosphere at 300 mTorr at RT. These conditions for the Pd films were previously shown to lead to nanoporous films, which are beneficial for SAW sensing of hydrogen [15]. After the deposition of WO_3_ and Pd/WO_3_ films, the resonant SAW frequency downshifted by 530 and 550 kHz, respectively, from the oscillation frequency with no film. The laser energies of about 75 mJ used for Pd were lower than those for WO_3_ due to the lower ablation threshold for the metal.

The film morphology was analyzed by using scanning electron microscopy (SEM, FEI QUANTA and Thermo scientific Apreo SEM (Thermo Scientific, Waltham, MA, USA)). The crystalline structure of the films was determined by X-ray Diffraction (XRD, X’Pert Panalytical, Almelo, Netherlands).

The SAW sensor was designed as a two-port resonator fabricated on a ST-X cut quartz substrate, which has good temperature stability at RT compared to other commonly used piezoelectric substrates such as LiNbO_3_ or LiTaO_3_. The 200 nm thick gold interdigital transducers (ITDs) were produced by standard photolithographic techniques and were deposited on a 10 nm chromium layer, which ensured good adhesion to the quartz substrate. A double-double finger design was used with 50 electrodes pairs and a periodicity of 11 µm. The SAW sensors with an oscillating frequency of 69.5 MHz were designed in a double-double configuration with 50 electrodes pairs, a 2500 µm acoustic aperture, and 45.2 µm wavelengths. The quartz dimension was 38 mm × 10 mm and cut in a parallelogram geometrical configuration with a 45° angle in order to reduce the reflection of acoustic waves on the edge of the substrate. The active area where the sensitive layer was deposited by PLD between ITDs was 10 mm^2^. A frequency counter (Pendulum CNT-91, Pendulum Instruments AB, Stockholm, Sweden) connected to a computer system with a Time View III software monitored the frequency change.

All the measurements were made at RT using mass flow controllers. Two cylinders were used one with a hydrogen gas mixture (2% H_2_/98% synthetic air) and another with synthetic air (100%). The hydrogen concentration in the SAW sensor testing box was controlled by varying the flow rate from the gas cylinders and keeping the total rate constant at 0.5 m^−1^ for all the measurements.

## 3. Results

### 3.1. Film Morphology and Structure

**Figure 1:**
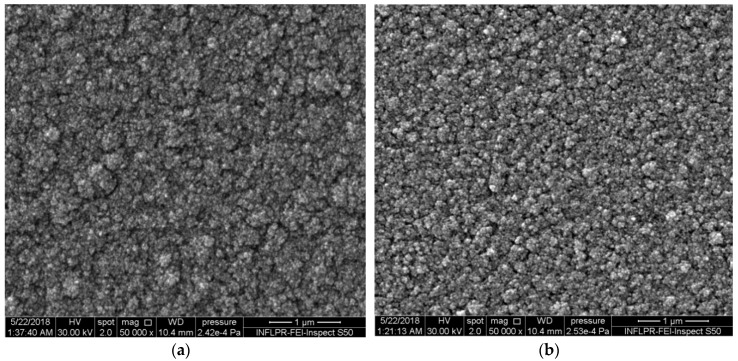
SEM images of the surfaces of WO_3_ thin films deposited in 500 mTorr O_2_ at different substrate temperatures. (**a**) 300 °C and (**b**) 600 °C.

WO_3_ films deposited in vacuum at room temperature have a metallic shine, which confirms the fact that the absence of an oxygen atmosphere does not allow the formation of stoichiometric tungsten oxide. The WO_3_ films deposited in an oxygen atmosphere have a thickness of about 620 nm, which is measured in section by SEM. In 150 mTorr oxygen or more, at a flow of 1 sccm, the WO_3_ layer is transparent, which indicates the presence of stoichiometric oxide and is confirmed by XRD. SEM results indicate the dependence of the film morphology on the deposition temperature and oxygen pressure. At relatively high deposition pressures of 500 mTorr O_2_, the films consist of loosely packed agglomerations of nanoparticles for both 300 °C and 600 °C (Figure 1). The difference between the two temperatures at this pressure is that the nanoparticle agglomerations visible in SEM images appear to be larger at 300 °C than at 600 °C. However, the films are porous at both temperatures and this leads to similarities in their sensing properties, which will be discussed later.

**Figure 2:**
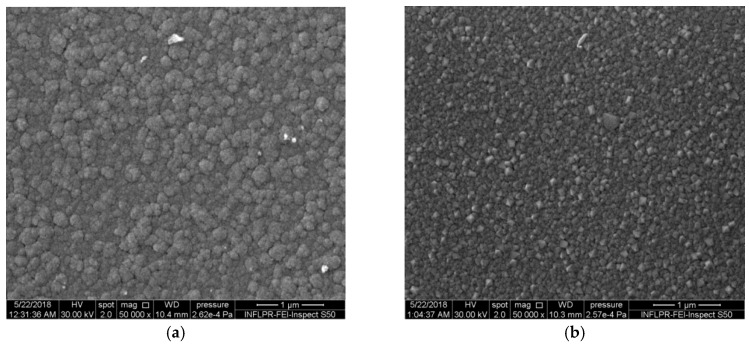
SEM images of the surfaces of WO_3_ thin films deposited in 150 mTorr O_2_ at different substrate temperatures. (**a**) 300 °C, (**b**) 600 °C.

**Figure 3:**
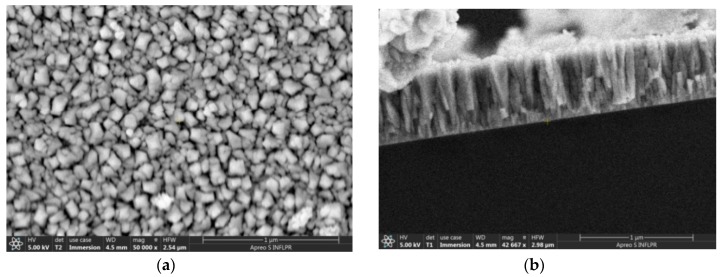
SEM images of the surfaces of Pd/WO_3_ bilayer thin films. The WO_3_ layer is deposited at 600 °C in 150 mTorr O_2_. (**a**) Surface. ( **b**) Section.

**Figure 4:**
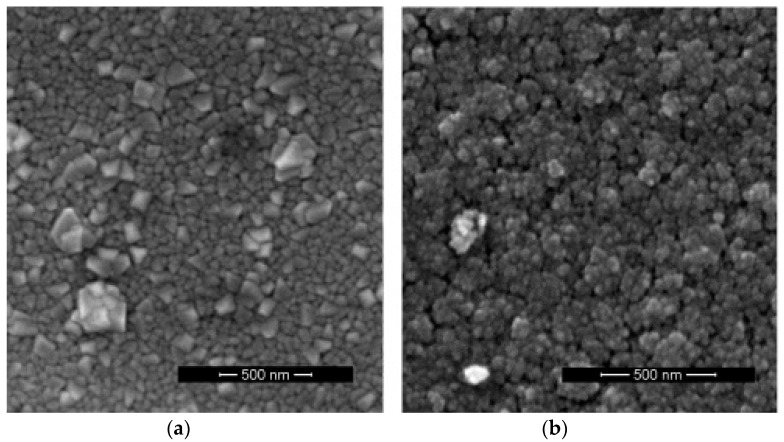
SEM images of the surfaces of WO_3_ thin films deposited in 300 mTorr O_2_ at different substrate temperatures. (**a**) 600 °C; (**b**) 300 °C.

At lower deposition pressures, the difference between the morphologies of the WO_3_ films deposited at different temperatures is evident (Figure 2). The films deposited at 300 °C and 150 mTorr have relatively densely packed nanoparticle agglomerations. Those deposited at 600 °C and 150 mTorr oxygen clearly present a columnar morphology, which is visible both on the surface and in the cross-section (Figure 2b and Figure 3). The nano-columns have dimensions of about 40 to 90 nm and extend through the thickness of the film. The fact that the nanocolumns are spaced apart, as can be seen in Figure 3a, is favorable for the diffusion of hydrogen into the film. At the intermediate pressure of 300 mTorr, while the films deposited at 300 °C still have rounded nanoparticle agglomerations and a porous nanostructure, those deposited at 600 °C already present the angular structure typical of nanocolumns (Figure 4).

The Pd films deposited on top of the WO_3_ layers, being thin (about 20 nm), do not affect the overall morphology of the film. In particular, the columnar nanostructures visible in Figure 2b, which is a SEM image of a WO_3_ film, also appear in Figure 3a (an image of a Pd/WO_3_ film). The nanostructure of the films has a considerable influence on the SAW sensor properties, which we will discuss below.

**Figure 5:**
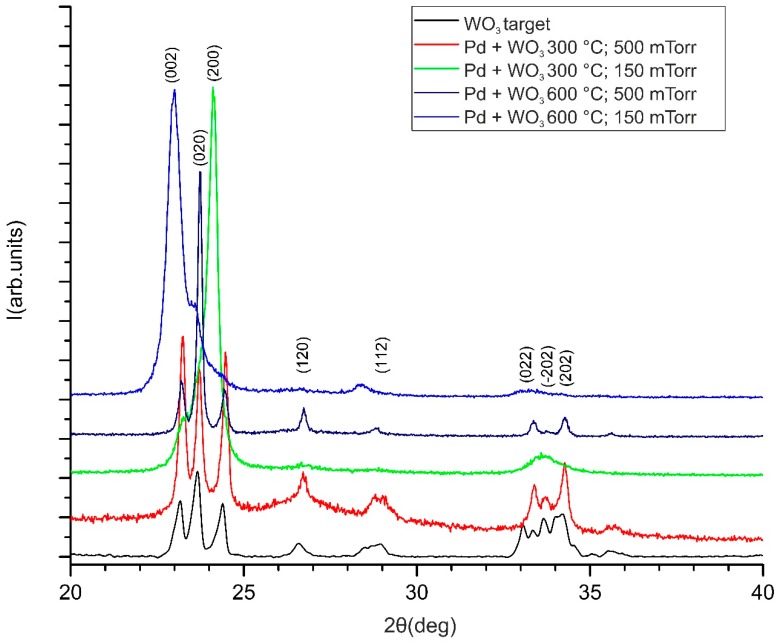
XRD patterns of the WO_3_ target and the Pd/WO_3_ sensitive films deposited in various temperature and pressure conditions.

It is known that the interaction of WO_3_ with H_2_ depends on the crystallinity of WO_3_, which is proven by in situ Raman studies of H_2_ interaction with WO_3_ films [16]. However, no studies on the effect of preferred crystal plane orientation growth of WO_3_ sensitive films on sensing properties have been reported for SAWs. XRD characterization of our deposited films indicates differences in the orientation for films deposited in different temperature and pressure conditions (Figure 5). Since the Pd films are relatively thin (about 20 nm) and is deposited in conditions that ensure a porous nanostructure, as previously discussed. Pd peaks are not present in the XRD data and the spectra of Pd/WO_3_ films are not different from films containing WO_3_ alone. The diffraction peaks for all the XRD patterns presented in Figure 5 could be indexed to monoclinic-WO_3_ (ICDD card No.043-1035). Different preferential orientation growth is visible by the relative intensities of the characteristic monoclinic major reflections (002), (020) and (200). It is interesting to note that texturing appears not only for the sample which shows nanocolumnar growth in SEM images (WO_3_ layers deposited at 600 °C and 150 mTorr oxygen), but also for other films. The degree of orientation for each film could be revealed by estimating the fraction of the intensity of each peak from the sum of the intensities of the other three (Table 1). All three peaks are of approximately equal intensities in random orientation. As expected, the film with a nanocolumnar structure exhibits preferential orientation along the (002) direction and the c-axis orientation, as reported previously for WO_3_ thin film deposition [9,17]. The WO_3_ film deposited at the lower 300 °C temperature and 150 mTorr also shows preferential orientation but along the (200) plane direction. The film obtained for 600 °C and 500 mTorr, although showing nanoporosity in SEM images (similar to 300 °C and 500 mTorr), presents a preferential (020) orientation. Both films deposited at a lower pressure have their dominant peaks shifted towards lower angles and, hence, to larger lattice parameters and unit cell volumes in Table 1. In our samples, random orientation is present only in the case of the WO_3_ sensitive film obtained at 300 °C and 500 mTorr. Although studies of the dependence of crystalline orientation on deposition conditions are described in literature [6,18], we have not found reports of (200) and (020) planes grown in WO_3_ films used in the sensors.

### 3.2. Sensor Properties

**Figure 6:**
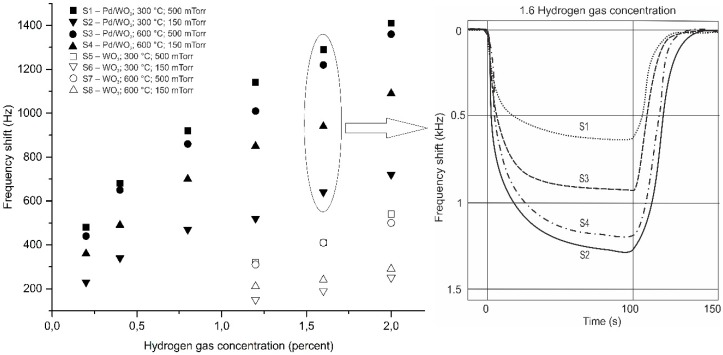
(**left**) Frequency shifts of the WO_3_–based sensors (open symbols) and Pd/WO_3_-based sensors (closed symbols) for various hydrogen gas concentrations. (**right**) Example of Pd/WO_3_ sensor response towards 1.6% H_2_ gas concentration at RT.

The frequency shift of the SAW sensors based on the sensitive films described above for various H_2_ concentrations is presented in Figure 6. All of the sensor properties reported are obtained at room temperature. Repeating 10 measurements of the frequency deviation for each of the four sensor films yielded errors below ± 3.5%. In all cases, the sensor response increases with hydrogen concentration. No sign of saturation of the frequency shift appears at the concentrations we investigated (which are all well below the 4% LEL–lower explosive level [19]). The results obtained for sensitive films consisting in WO_3_ only (open symbols) show a much poorer response than those with Pd/WO_3_. No sensor response can be obtained below 1.2% hydrogen. The frequency shift, in this case, is larger for the WO_3_ porous layers deposited at high oxygen pressures [20]. Although both SEM images and XRD analysis indicate differences between WO_3_ layers deposited at different temperatures and the same pressure, their sensing properties are practically identical.

When the thin Pd layer is deposited on top of WO_3_, the sensing properties of the SAWs improve considerably (Figure 6, full symbols). Responses are obtained for lower hydrogen concentrations and the frequency shifts are much larger than in the prior case. As in the case of the WO_3_-only sensing layer, the nanoporous films deposited at the high oxygen pressure have the largest frequency shifts due to a large effective surface area. These shifts do not depend on whether the deposition temperature is 300 °C or 600 °C. The most interesting result obtained is that the sensor properties of the film based on WO_3_ was deposited at the highest temperature (600 °C) and the lower pressure (150 mTorr) increased more when a Pd layer was added relative to the other sensors.

Table 2 presents the sensitivity and LOD (limit of detection defined as three times the noise level divided by the sensitivity) for the SAW sensors with sensing films deposited in different conditions. The sensor sensitivity, given in the table, was determined from the slope of the linear curve-fit of the data in Figure 6. The noise level was around 40 Hz for all the sensors and was determined by measuring the resonance frequency for 10 min as a maximum frequency deviation from the trend line (best fit line). The results presented in Table 2 confirm that the best sensor properties are those of the porous bilayer Pd/WO_3_ films (S1 and S3) and that the bilayer films with a nanocolumnar WO_3_ layer structure (S4) have a higher sensitivity and a lower limit of detection than the one with a dense WO_3_ structure (S2).

In conclusion, the sensor properties depend to a certain extent on the morphology and crystallinity of the WO_3_ layer. The presence of the top Pd layer improves the sensor response considerably, which was previously reported [7,8]. In comparison to dense Pd/WO_3_ bilayer-based SAWs, our nanostructured Pd/WO_3_ films can lead to a better sensor response even during an RT operation. For dense layers, frequency shifts of the order of tens of Hz are obtained (at temperatures above RT) for 2% hydrogen [10] while, in all of the Pd/WO_3_-based sensitive layers, the frequency shifts are 1 to 2 orders of magnitude larger at the same H_2_ concentration at room temperature. By optimizing the WO_3_ layer thickness, Hejczyk and Urbanczyk obtained very good hydrogen sensing results at RT [14]. While we have not optimized the thickness of the sensing layers, we have obtained results comparable to theirs.

## 4. Discussion and Conclusions

The morphology of the laser-deposited thin films depends on the deposition conditions (temperature and pressure) and can easily be controlled in PLD. At high deposition pressures (500 mTorr oxygen), the deposition temperature has little influence on the morphology of the WO_3_ layers. At these pressures, the species emitted from the target undergo many collisions with the gas species until they arrive at the substrate, which they reach with relatively small energies. The substrate temperatures used in our depositions cannot compensate these energies and the arriving species cannot migrate across the surface to lead to preferable arrangements. In addition, agglomerations of nanoparticles form in the target-substrate region at such pressures and such agglomerations are too massive to be altered by the substrate heating. Although there are differences in the crystalline structure, these appear to play no role in the sensing properties. At low WO_3_ deposition pressures (150 mTorr oxygen) where the energy of the species arriving at the substrate surface is larger than in the former case, the temperature of the substrate plays a role in the arrangement of the incoming species on the substrate surface. Thus, at the higher 600 °C temperature, nanocolumnar growth is possible.

The XRD results indicate that preferred orientation growth is present in the WO_3_ films not only in the case of the columnar growth shown in SEM images, but for other deposition conditions including those that lead to porous films. Such texturing may be explained by the strain, which appears in the films during the growth process. However, it is only the predominantly c-axis growth, which leads to the formation of nano-columns and seems to affect the sensing properties of the films.

The thin Pd layer deposited on top of WO_3_ is essential for obtaining a good SAW sensor response to H_2_ even though the morphology and crystalline structure of WO_3_ is practically unchanged by the deposition of a Pd layer on top. Pd efficiently dissociates H_2_, which generates H^+^ ions and electrons that diffuse into the WO_3_ layer at a rate that depends on its morphology and crystalline structure [7,16]. In the absence of Pd, the SAW sensor properties are weak even though porous WO_3_ layers deposited at high pressures give somewhat better results than less porous ones. The SAW sensor response is greatly improved by Pd, but the improvement depends on the morphology of the WO_3_ layer. This is a result that we have not found previously. The most interesting result obtained is that the sensor properties of the film based on WO_3_ deposited at the high temperature and lower pressure are improved much more by the added Pd layer compared to the improvement of other WO_3_ layers. This could be explained by the fact that the nanocolumnar structure promotes the diffusion of the hydrogen into the WO_3_ once it has been dissociated in the Pd layer. In the absence of the Pd layer, the WO_3_ nanocolumns do not facilitate diffusion of H_2_ to a greater extent than the relatively dense structure obtained at the lower deposition temperature of 300 °C. However, the frequency shifts obtained for the nano-porous WO_3_-based bilayer films are still higher than those for the nanocolumnar WO_3_ films.

An additional mechanism is involved in bilayer Pd/WO_3_ SAW sensing films. It is well known that surface acoustic wave propagation is very sensitive to changes in electrical conductivity through acoustoelectric interactions [21]. Acousto-electrical interactions between the electrical potential accompanying SAW propagation and mobile charges in the sensing film can be enhanced in bilayers [12,17]. For example, bilayer Pd/WO_3_ films have better results than WO_3_ films in SAW in the case of dense films [10]. Our Pd/WO_3_ bilayers have different morphologies and better hydrogen sensing properties at RT than the dense ones even though we have not optimized layer thicknesses, which was described in Reference [10]. In hydrogen sensing, since the dissociated H_2_ molecules lead to the formation of H^+^ ions and electrons, which diffuse into the WO_3_ lattice [8,16], this will lead to changes in the acoustoelectric interaction between the surface acoustic wave and the space charges in the sensitive material. In addition, the oxygen vacancies in the laser-deposited WO_3_ film, which depend on oxygen deposition pressure (as studied in the case of resistive sensors in Reference [17]) will also affect the acoustoelectric interaction in the case of SAW sensors. For bilayers, the mobile charges generated in both component layers interact with the traveling electrical potential associated with the SAW, which leads to changes in the wave velocity. Therefore, the morphology and structure of the Pd and WO_3_ components of the sensitive layer can affect the interaction of H_2_ with the SAW sensor in a complicated way.

The frequency shifts obtained for hydrogen concentrations well below LEL compare favorably with other H_2_ sensors based on WO_3_ including SAW as well as resistive and gasochromic-based ones [7,8,9,10]. An important point is that all our results are obtained at room temperature while most other Pd/WO_3_ sensors report the operation above RT. Similarly, Pt/WO_3_-based SAW sensors also require operation at 100 °C or more for good sensing properties [12]. Since optimization of the WO_3_ layer thickness has been proven to lead to the improvement of sensor properties for dense films [14], we intend to direct future research along this direction.

Our results on the dependence of SAW sensor properties on the morphology and crystalline structure of Pd/WO_3_ bilayers could prove applicable to other materials, which have a tendency to the nano-column growth and are used in sensor applications such as ZnO or SnO_2_ [9,22,23]. The results could also lead to the improvement of a WO_3_-based SAW sensor response to other gases such as NO or CO.

## Figures and Tables

**Table 1:** Structural information of the Pd/WO_3_ sensing films deposited in various conditions. The standard sample is WO_3_ powder, ICCD card No.043-1035. The WO_3_ target is a pressed pellet. The deposition conditions for the films are: S1 300 °C/500 mTorr O_2_, S2 300 °C/ 150 mTorr O_2_, S3 600 °C/500 mTorr O_2_, and S4 600 °C/150 mTorr O_2_.

Samples	Unit Cell Parameters					Preferential Orientation			
	a (Å)	b (Å)	c (Å)	β (^o^)	Vol Å^3^	I_002_/Σ_I_	I_020_/Σ_I_	I_200_/Σ_I_	
WO_3_-Powder	7.297	7.539	7.688	90.91	422.8	0.330	0.326	0.344	random
WO_3_-Target	7.290 (4)	7.537 (6)	7.682 (6)	90.88 (1)	422.03	0.280	0.450	0.270	random
Films									
S1	7.25 (2)	7.52 (1)	7.69 (2)	91.17 (4)	419.04	0.371	0.298	0.330	random
S2	7.26 (2)	7.53 (3)	7.69 (2)	91.01 (4)	420.19	0.090	0.193	0.716	a-axis
S3	7.325 (8)	7.522 (4)	7.672 (9)	90.57 (1)	422.68	0.139	0.751	0.109	b-axis
S4	7.359 (5)	7.541 (6)	7.733 (6)	91.42 (2)	428.44	0.792	0.155	0.052	c-axis

**Table 2:** Sensitivity and limit of detection (Δf-frequency change, c–hydrogen gas concentration) for the various sensitive films.

Sensor Type	Sensitivity (Δf/c) (Hz/ppm)	LOD (ppm)
S1—Pd/WO3; 300 °C; 500 mTorr	0.13	4540
S2—Pd/WO3; 300 °C; 150 mTorr	0.06	9770
S3—Pd/WO3; 600 °C; 500 mTorr	0.12	4710
S4—Pd/WO3; 600 °C; 150 mTorr	0.1	7700
S5—WO3; 300 °C; 500 mTorr	0.03	1120
S6—WO3; 300 °C; 150 mTorr	0.01	2270
S7—WO3; 600 °C; 500 mTorr	0.03	1190
S8—WO3; 600 °C; 150 mTorr	0.02	1490
